# Beyond boundaries: how autonomy and technology promote work engagement and well-being in remote workers

**DOI:** 10.3389/fpubh.2025.1659185

**Published:** 2025-09-08

**Authors:** Ilaria Olivo, Valerio Ghezzi, Ivan Marzocchi, Luigi Fusco, Francesca Spinella, Stefano Isolani, Matteo Ronchetti, Monica Ghelli, Simone Russo, Claudio Barbaranelli, Benedetta Persechino, Sergio Iavicoli

**Affiliations:** ^1^Department of Psychology, Sapienza University of Rome, Rome, Italy; ^2^INAIL-Department of Occupational and Environmental Medicine, Epidemiology and Hygiene, Rome, Italy; ^3^Ministry of Health, Rome, Italy

**Keywords:** remote work, working conditions, employee well-being, public administration, longitudinal study

## Abstract

This longitudinal investigation explores the role of technology as resource and job autonomy in influencing remote workers’ engagement and private lives during the transition to remote work, spurred by the COVID-19 pandemic. Data of 194 employees of the Italian Public Administration were collected across three time points: pre-remote work (T0, December 2018), limited remote work (T1, December 2019), and full-time remote work during the pandemic (T2, July 2020). The findings showed that job autonomy and technology as resources enhance work engagement, while engaged workers develop a more positive perception of technological tools. Notably, engagement did not predict autonomy, likely due to contextual constraints such as the abrupt transition to full-time remote work. The perception of technology as a resource significantly improved private life outcomes. These insights underline the critical role of tailored technological support and organizational policies in fostering a productive and balanced remote work environment, enabling organizations to better meet the needs of their workforce.

## Introduction

Remote work refers to any work carried out outside the employer’s premises regardless of the technology used (1). Due to the pandemic, remote work has become an increasingly prevalent and necessary practice (2). As a result, organizations were faced with an emergency state, and they had to provide employees with technological resources to do their work remotely (3). This introduction has led to a radical and abrupt change in the work arrangement, with workers being required to adapt to a new way of working from home, in most cases without any prior preparation or adaptation phase (2). The effects of this change (as well as their boundary conditions) still need to be clearly understood, as it may lead to both positive and negative outcomes (4). Specifically, several studies emphasized the positive aspects of remote work, highlighting the increased flexibility, reduced commuting time and costs, and improved work-life balance (3, 5–7). These can increase satisfaction with one’s work, especially after an initial period in which remote working experience could have been traumatic (8). However, some studies suggest that remote work can be detrimental to well-being; for example, the lack of social contact could increase the perception of loneliness in remote workers (9).

The present study focused on a specific group of employees from an Italian Public Administration, which increased the number of remote working days per week over the past 3 years. Drawing on the Job Demands-Resources model (10), the present research aims to assess the influence of remote work and the use of technology on workers’ private lives and work engagement over time.

## Literature review and hypothesis development

Since the beginning of the 21st century, technological progress and innovation has profoundly changed how we communicate and live. Technologies such as tablets and mobile devices are not just helping people to do things better and faster, they are enabling profound changes in how work is done in organizations. Indeed, the digital transformation and the transition to Industry 4.0 have brought about significant challenges and changes for organizations and workers, resulting in the reorganization of job design (11).

This trend rapidly increased due to the COVID-19 pandemic, when some governments introduced an obligation for companies to enable their workers to work remotely, mostly from home (12). The outbreak of the pandemic had significant implications for workers worldwide: governmental lockdowns and work-from-home arrangements forced many employees to adjust their work routines. Organizations were forced to implement remote work arrangements to ensure business continuity. As a result, remote work has become a critical work arrangement for companies to cope with the effects of the pandemic emergency (13). In Europe, the proportion of remote workers increased from 11% before to 48% during the pandemic (14). Italy was the European nation with the lowest percentage of remote workers in 2020, but the number of remote workers increased by 69% due to the pandemic outbreak (14, 15).

Remote work has been an option for several decades, but it has gained more significance recently, owing to the changes in technology and the new normality resulting from the pandemic. Many jobs across various sectors have been created or evolved for remote work, such as marketing, sales, advisory services, customer service, education, digital services, computing, or social networks. In this vein, remote work might represent a development opportunity for everyone’s job, particularly for those facing mobility difficulties due to distance, resources, age, or disability (16). More and more people are working from home, regardless of their equipment and environment, posing new challenges to organizational managements, which are required to implement effective practices and policies to ensure remote workers have a healthy and fulfilling work environment. One example of such practices is improving tools for sharing information and documents. In situations where changes are introduced within the organization, clear communication and good sharing from management have been found to boost job satisfaction. On the other hand, poor management of change can have a negative impact on workers’ well-being (17).

Although some studies highlighted the risks associated with remote work, such as the lack of social interaction, irregular work hours affecting the quality of sleep, and the interruption of work obligations due to home activities (18, 19), other studies suggested that employees can benefit from remote work by providing them with increased job autonomy and by reducing their commuting frequency (20, 21). Furthermore, Peters et al. (22) found that teleworking can increase work engagement by increasing job autonomy, reducing stress, and enhancing job satisfaction. Indeed, remote workers have typically greater control over their time, space, and pace of work, providing greater flexibility and the ability to adapt work to their needs. This can lead to a better balance between work and personal life (23, 24) and help workers balance their work and personal responsibilities, improving their quality of life, and reducing stress levels (25). Thus, organizations should properly introduce remote work as a valuable resource for workers, allowing them to complete their tasks with greater freedom and flexibility. Moreover, remote work arrangements limit job interruptions, allow for closer collaboration with clients and colleagues in a virtual environment, and provides the option to work from informal locations, which can increase confidence and productivity (26), allowing workers to adapt their work activities to their needs and preferences (27). This can result in more efficient and effective work, leading to better performance (28).

According to the Job Demands-Resources model (JD-R) (29), job resources are defined as “those physical, psychological, social, or organizational aspects of the job that are either/or: functional in achieving work goals; reduce job demands and the associated physiological and psychological costs; stimulate personal growth, learning, and development” [(30), p. 312]. Job resources can be found at different levels, such as organizational, interpersonal, social relationships, and job design. For instance, autonomy is an example of an organizational-level resource (10).

Job autonomy is considered one of the most important job resources (31). It refers to the extent of control and freedom a worker possesses to organize and perform his/her work activities (32), increasing his/her sense of responsibility and control (33). In this sense, Bošković (34) found that autonomy positively impacts employee engagement in the digital environment. Workers’ ability to decide on the manner, place, and time of performing tasks increases their perception of the meaningfulness of their work. Moreover, autonomy encourages creativity and makes it easier to adapt to change (35). According to motivational process of JD-R model (30), job resources (i.e., job autonomy and technological tools) can fulfil fundamental psychological needs, such as the desire to belong and the desire to engage in meaningful activities in an independent manner (36).

Thus, we hypothesise that:

H1: Job autonomy will positively sustain work engagement over time.

H2: The perception of technology as a resource will positively sustain work engagement over time.

According to recent refinements of Bakker and colleagues’ JD-R model (2023), work engagement can be considered both an outcome and a predictor of proactive behavior. Indeed, the JD-R model proposes that workers may experience a positive cycle in which they become more engaged and motivated to optimize their work. This is achieved through vigor, dedication, and absorption towards one’s work, which are all aspects of work engagement. As they become more engaged, employees may proactively seek to improve their jobs by increasing resources and optimizing demands. If this positive gain cycle continues, it may even result in a gain spiral, where the relationships between work engagement, proactive behavior and resources become more and more positive, leading to even higher levels of these variables [see also (37)]. According to Salanova et al. (38), “gain spirals” is a term used to describe the phenomenon where employees who have acquired resources tend to experience further gains more easily. This group finds it easier to offset any loss of resources and orchestrate additional gains. As a result, they tend to experience higher levels of resource gains overall (37). Therefore, workers who are engaged in their work tend to have an innate motivation to stay engaged and are proactive in optimizing their job roles. This can involve increased resources and more balanced demands aimed at improving their work experience. Over time, employees shape their job responsibilities, which generates job and personal resources to manage job demands and cultivate future work engagement (10).

Thus, we hypothesise that:

H3: work engagement will positively influence job autonomy over time.

H4: work engagement will positively influence the perception of technology as a resource over time.

Introducing advanced technology has made remote work smarter, enabling greater control over tasks and time management, leading to improved psychological well-being (39). This increased flexibility would allow employees to dedicate more time to other activities, such as engaging in physical activities and dedicating time to their relatives and leisure (40). Remote work has the added benefit of saving time that would otherwise be spent commuting to and from the office. This extra time can be used to pursue other activities and priorities, leading to a more balanced and fulfilling lifestyle for remote workers (41). Developing effective strategies for remote work includes establishing a structured home environment, setting clear boundaries between work and personal life, and maintaining regular communication with colleagues using technological tools [e.g., virtual platforms; see (42)]. By practicing this approach, workers can take a break from their regular work environment to better manage their work-life balance, resulting in a healthier lifestyle. This, in turn, can lead to improved physical and mental well-being (25).

Thus, we hypothesise that:

H5: The perception of technology as a resource would positively predict improvements in workers’ private lives.

H6: Job autonomy would positively predict improvements in workers’ private lives.

As previously illustrated, having the autonomy to make decisions and control over his/her own work is a significant determinant of employee engagement (34), and being engaged at work is essential to taking the initiative and persisting through complex tasks (10). Engaged workers are also more open to new experiences (43). As a result, they tend to explore their environment and are more prone to accept changes positively. Innovative behavior is another positive consequence of experiencing positive affect and work engagement (44). These findings suggest that work engagement can broaden workers’ horizons, making them more likely to explore alternative and innovative paths.

Research showed that engagement has several positive implications not only in work-related aspects but also in personal life aspects. Rodríguez-Muñoz et al. (45) found that workers were happier in their private lives (e.g., with their partners) when they experienced higher work engagement. Moreover, work engagement positively relates to work-to-family facilitation and personal happiness (46). Finally, from the positive psychology perspective, work engagement can contribute to individuals’ needs to lead a life fulfilled in all its aspects (47). Therefore, the workers who become highly engaged tend to feel more satisfied.

Thus, we hypothesise that:

H7: Work engagement will mediate the relation between job autonomy and the perception of technology as a resource.

H8: The perception of technology as a resource will mediate the relation between work engagement and Improvement in private life.

## Materials and methods

### Participant and procedure

This study is part of a project on remote work developed by the Italian Workers’ Compensation Authority (INAIL) and called “Smart Working in INAIL,” which includes 319 workers that have been selected from the Human Resource Department from different organizational units. Inclusion criteria were based on the adaptability of the work activities to a flexible work modality and the workers’ skills in using some ICTs offered by the organizations. Most of the workers included in this project had administrative and technical positions and were employed in operational activities. Participants filled out an online-based questionnaire containing questions about the use of new information and communication technologies, their perception of job autonomy, their perceptions about the Improvement in their personal lives due to the introduction of remote work, and work engagement. All the data were collected three times. The first wave (Time 0) aimed to collect information up to 1 month before the beginning of the remote working period (December 2018). The second wave (Time 1) aimed to get information about the changes that could have arisen after 1 year of remote work, 1 day per week. The third wave (Time 2) aimed to get information about the changes that could have arisen after the COVID-19 pandemic outbreak and remote work 5 days per week. Participants were invited to participate in the study through an email from the organization.

Of the 319 workers invited to the study, 273 took part in the first wave in December 2018, with a response rate (RR) of 74.76%. All the participants of Time 0 were invited to compile the Time 1 questionnaire. Of them, 198 answered to the second part of the study (RR = 83.54%), in December 2019. Again, all the workers who participated in Time 1 were invited to fill out the Time 2 questionnaire. Of them, 194 took part in the study’s final wave (RR = 97.98%) in July 2020.

The final sample consisted of *n* = 194 workers from INAIL with a mean age of 52.40 (*SD* = 6.29), and most of them, 62.4% (*n* = 121), were female. 31.4% (*n* = 61) of the respondents reported not having children, 13.4% (*n* = 26) having one child, 10.3% (*n* = 20) having two children, and 44.8% (*n* = 87) did not answer this question. Almost all the participants (75.8%, *n* = 147) had a permanent contract, while only one participant (0.7%) had a fixed-term contract, and 23.7% (*n* = 46) did not answer this question. Most of the workers (73.6%, *n* = 148) had a full-time job, while the 23.7% (*n* = 46) did not answer this question. Most of the sample (63.9%, *n* = 124) was represented by workers with an organizational tenure of more than 15 years, 4.6% (*n* = 9) from six to 10 years, 3.6% (*n* = 7) between 11 and 15 years, 3.1% (*n* = 6) between one and 5 years, and 24.7% (*n* = 48) did not report their organizational tenure. Sample characteristics are reported in Table 1.

**Table 1:** Sample characteristics.

Variables	M / N	*SD* / %
Age	52.40	6.29
Gender		
Male	27	13.9
Female	121	62.4
nr	46	23.7
Children		
0	61	31.4
1	26	13.4
2	20	10.3
nr	87	44.8
Contract		
Permanent	147	75.8
Fixed term	1	0.5
nr	46	23.7
Working hours		
Full-time	148	76.3
nr	46	23.7
Organizational tenure		
1 to 5 years	6	3.1
6 to 10 years	9	4.6
11 to 15 years	7	3.6
More than 15 years	124	63.9
nr	48	24.7

M, mean; SD, Standard Deviation; nr, not resonded.

### Measures

#### Technology as a resource (TR)

We used a 9-item scale developed *ad-hoc* for the present study, which assessed the perception of workers about ICT offered by the organizations as support for their work through a 5-point Likert scale ranging from 1 (Strongly disagree) to 5 (Strongly agree). The measure investigated two main areas: (a) *ICT tools as a support for improving work activities* (e.g., “The technological tools provided help to improve the quality of my work”); and (b) *ICT tools as a support for improving relationship between colleagues* (e.g., “I believe that the new technological tools provided for work can promote interaction with my colleagues”). The relatively high correlation (1.00) between the factor scores derived from the two-factor solution and the scores computed by summing all the items suggests that the facets we used can be regarded as proper indicators of the latent factor [see (48)]. Thus, the total score scale was used in this study. The McDonald’s Omega reliability coefficients for the total scale were ω_T0_ = 0.91, ω_T1_ = 0.92, ω_T2_ = 0.93.

#### Autonomy (AUT)

We used 3 items from the Management Standards Indicator Tool^1^ [MS-IT; (49)] which assessed the perceived autonomy in the management of their work (e.g., “I have a choice in deciding how I do my work”) through a 5-point Likert scale ranging from 1 (Strongly disagree) to 5 (Strongly agree). The reliability coefficient in this study was ω_T0_ = 0.80, ω_T1_ = 0.83, ω_T2_ = 0.85.

#### Work engagement (ENG)

We used 3 items from the Ultra-Short Measure for Work Engagement (50), which assessed workers’ vigor, absorption, and dedication to their work (e.g., “I am enthusiastic about my job”) through a 7-point Likert scale ranging from 0 (Never) to 6 (Always). The reliability coefficient in this study was ω_T0_ = 0.76, ω_T1_ = 0.82, ω_T2_ = 0.81.

#### Improvements in private life (IPL)

We used one item which assess participants’ perception of improvements in their private life due to remote work (“Remote work has improved my personal life”) through a 5-point Likert scale ranging from 1 (Not at all) to 5 (Completely). This variable was measured only in T1 and T2; in T0, none of the workers was in a remote work condition.

#### Covariates

We included gender (1 = male, 2 = female) and the presence of children (1 = yes, 2 = no) as covariates in the main analysis set.

### Data analysis

As a preliminary analysis, we ran an attrition and missing data analysis using a multifaceted approach (51). Firstly, we performed Little’s test (52) to assess whether the collected data were missing completely at random (MCAR). Secondly, we ran a series of Multivariate Analyses of Variance (MANOVA) to understand if attrition between adjacent assessment time points and from T0 to T2 was partially selective. For this purpose, we created a dummy variable (0 = non-attrited; 1 = attrited participants) as a between-subjects factor. Thirdly, we checked the homogeneity of covariance matrices between attrited and non-attrited subjects through the Box’s M tests (53).

Longitudinal invariance was used to evaluate the different measurement properties of the study measures across the three-time points of assessment. We precisely followed Meredith’s approach (54). Firstly, we tested an unconstrained model (i.e., *configural in*var*iance*) in which no constraints across time were imposed on any of the parameters (i.e., the same factor and the same patterns of fixed and freed parameters). Secondly, we constrained factor loadings to be equal across the different waves (i.e., *metric in*var*iance*); thirdly, we constrained observed intercepts (i.e., *strong in*var*iance*), and finally, we constrained residual variances (i.e., *strict invariance*) to be equal across waves. As these models are nested, we examined the tenability of the imposed constraints by calculating both Δ_χ2_ (Δ_df_) (55) and Δ_CFI_ tests. A non-significant restricted χ^2^ test with *α* ≥ 0.01 and Δ_CFI_ < 0.01 (56) supports the tenability of the imposed constraints and, thus, the longitudinal invariance.

Finally, the cross-lagged panel model with lag-2 effects [CL2PM; see (57)] was used to test the hypothesized paths among the variables. Compared to the classical CLPM, this model posited the higher-order autoregressive effect of T0 over T2 for all the study variables. The posited model included time-invariant gender and the presence of children as covariates (measured at T0) as control variables of all substantive measures. First, we implemented a model in which no constraints across time and effects were imposed on any parameter. Second, we constrained covariances between variables within each wave. Third, we constrained the autoregressive effects of each variable to be equal (i.e., from T0 to T1 and from T1 to T2). Fourth, we constrained cross-lagged effects between variables to be equal. Finally, we assessed the indirect effects underlying the relationships among the variables (58).

## Results

### Attrition and missing data analysis

Considering all the variables under study, Little’s MCAR test was non-significant (χ^2^_[14]_ = 17.32, *p* = 0.24), demonstrating that data were missing completely at random among the subjects included in the analysis. MANOVA results using T3 attrition as between-subjects factor and, as dependent variables, study variables at T1 (Wilk’s *Λ* = 0.985 F _(3,190)_ = 0.959 *p* = 0.41, partial η^2^ = 0.01) and at T2 (Wilk’s Λ = 0.973 F_(4,165)_ = 1.145 *p* = 0.34, partial η^2^ = 0.03) revealed no significant differences between attrited and non-attrited subjects at adjacent time points of assessment. Finally, all Box’s M tests were non-significant, suggesting that homogeneity of covariance matrices between attrited and non-attrited subjects held in each analysis. Finally, males were slightly more prone to drop the study at T1 after baseline (correlation between gender coded as 0 = male and 1 = female with T1 attrition was −0.15, *p* < 0.05), while other relevant socio-demographic variables were unrelated to missing data on the study variables. Overall, our results indicate that missing data were not a reason for concern in this study since the mechanisms underlying missing data resemble a combination of MCAR and MAR processes, which can be handled with full information maximum likelihood-based (FIML) estimators in further analyses.

### Descriptive statistics

Table 2 reports mean values, standard deviations, skewness, and kurtosis for the four study variables at T0, T1 and T2. Skewness and kurtosis were used to assess if scales meet univariate normality assumptions (59). Most of the scales exhibited non-problematic skewness and kurtosis levels since their values were between −1 and +1. However, skewness for *Work engagement* in T1 and T2 and kurtosis for *Technology as a resource (TR)* in T0, *Work engagement* in T1, *Autonomy* in T2, and *Improvement in private life* in T2 revealed a slight departure from univariate normality (53). Therefore, we run all the following analyses using M*plus* 8.10 and MLR estimator, as it corrects the standard error of model parameters for slight departures from univariate and multivariate normal distributions of the data (60).

**Table 2:** Descriptive statistics for the study variables.

Variables	M	*SD*	Skewness	Kurtosis	ω
T0					
TR	3.82	0.06	−0.58	1.93	0.91
AUT	3.74	0.68	−0.57	0.98	0.81
ENG	4.52	1.01	−0.46	0.08	0.75
IPL	na	na	na	na	-
T1					
TR	3.98	0.53	−0.06	0.09	0.91
AUT	3.72	0.71	−0.46	−0.08	0.83
ENG	4.56	1.15	−1.09	1.68	0.83
IPL	4.18	0.85	−0.84	0.50	-
T2					
TR	3.87	0.67	−0.28	0.40	0.93
AUT	3.70	0.73	−0.94	1.83	0.85
ENG	4.41	1.10	−0.67	0.28	0.81
IPL	4.16	0.96	−1.3	1.7	-

TR, Technology as a resource; ENG, Work Engagement; AUT, Autonomy; IPL, Improvement in private life; M, mean; SD, Standard Deviation; na, Not Assessed (IPL was not assessed in T0); ω coefficients were not computed for IPL as is a single-item measurement. T0 = Time 1, T1 = Time 2, T2 = Time 3.

### Longitudinal measurement invariance

Before running the cross-lagged model, we assessed the longitudinal invariance of each measure. Table 3 shows that for all the 3 scales considered (Technology as a resource, Autonomy, and Work engagement) all 3 levels of invariance were fully achieved.

**Table 3:** Longitudinal measurement invariance.

Invariance	χ2	*df*	p_χ2_	Δ_χ2_	Δ*df*	p_Δχ2_	CFI	TLI	RMSEA(p)	Δ_CFI_
TR										
Configural	15.27	15	0.43				1.00	0.999	0.010(ns)	
Metric	20.83	19	0.34	5.26	4	0.26	0.998	0.996	0.022(ns)	−0.002
Scalar	24.57	23	0.37	3.68	4	0.45	0.998	0.997	0.019(ns)	0.000
Strict	30.49	29	0.39	5.92	7	0.43	0.998	0.998	0.016(ns)	0.000
LMI	41.79	31	0.09	11.30	2	0.002	0.989	0.987	0.042(ns)	−0.009
AUT										
Configural	10.66	15	0.78				1.00	1.00	0.000(ns)	
Metric	10.73	19	0.93	0.72	4	0.95	1.00	1.00	0.000(ns)	0.000
Scalar	12.16	23	0.97	1.40	4	0.84	1.00	1.00	0.000(ns)	0.000
Strict	18.51	29	0.93	6.35	7	0.45	1.00	1.00	0.000(ns)	0.000
LMI	19.06	31	0.95	0.55	2	0.79	1.00	1.00	0.000(ns)	0.000
ENG										
Configural	14.72	15	0.47				1.00	1.00	0.000(ns)	
Metric	23.17	19	0.23	7.97	4	0.09	0.994	0.989	0.034(ns)	−0.006
Scalar	25.07	23	0.35	1.53	4	0.82	0.997	0.995	0.022(ns)	0.003
Strict	27.40	29	0.54	2.33	7	0.55	1.00	1.00	0.000(ns)	0.003
LMI	32.57	31	0.38	5,17	2	0.047	0.998	0.997	0.016(ns)	0.002

TR, Technology as a resource; ENG, Work Engagement; AUT, Autonomy; LMI, Latent Means Invariance.

After the inspection of modification indices, we found a slight increase between T0 and T1 in TR and a slight decrease between T1 and T2 in ENG. This size can be quantified, respectively, in 0.62 and 0.78 latent means.

### Cross-lagged panel model

**Figure 1:**
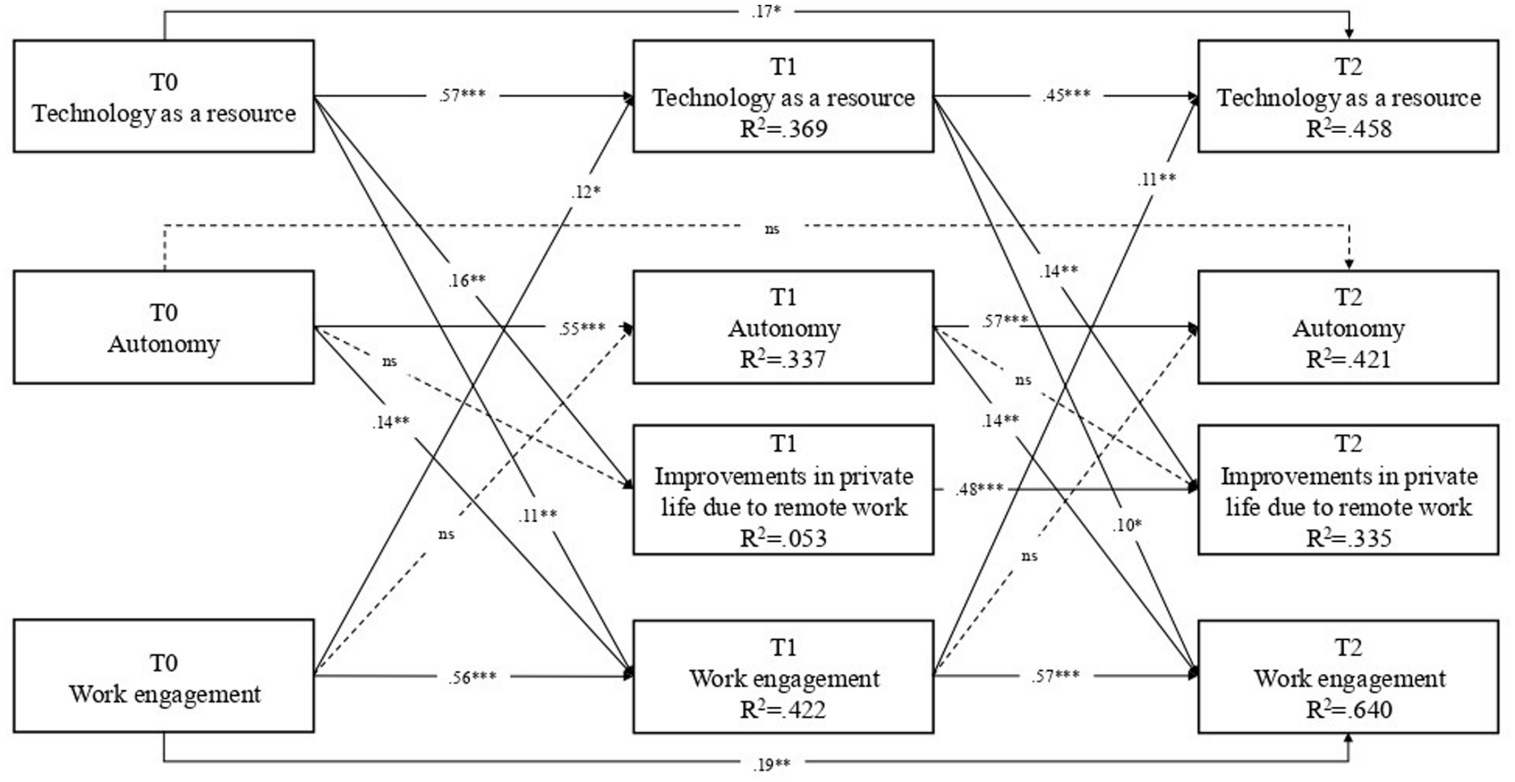
Cross-lagged panel model parameters. Results are completely standardized. All estimates were controlled for gender (1-males, 2 = f females) and number of children (0 = no 1 = yes). All variables within each wave were allowed to covary. The dotted lines indicate non-significant effects (*p* > 0.05); *** *p* < 001, *p* < 0.01,”“*p* < 05.

A cross-lagged with lag-2 effects panel model (CL2PM) has been used to test the main hypotheses. We compared two models, the first in which all autoregressive and cross-lagged relationships were unconstrained, the second in which these relationships were all constrained to be equal through time. The results showed that the full constrained model had a better fit (χ^2^_(26)_ = 31.607, *p* = 0.20, CFI = 0.99, RMSEA = 0.033 (90% CI 0.000–0.069, *p* = 0.742), SRMR = 0.05.) than the unconstrained model (χ^2^(17) = 20.468, *p* = 0.25, CFI = 0.99, RMSEA = 0.032 (90% CI 0.000–0.076, *p* = 0.698), SRMR = 0.04), so we accept the most parsimonious model which is the constrained one. Figure 1 reported the tested parameters in a completely standardized metric. Overall, all variables showed significant autoregressive paths across time points, suggesting that TR, AUT, ENG and IPL were consistently stable across contiguous waves.

In line with H1, results showed that AUT (T0) had a significant effect on ENG (T1) (*β* = 0.14, *p* = 0.003), and AUT (T1) had a significant effect on ENG (T2) (*β* = 0.14, *p* = 0.001). Similarly, in line with H2, TR (T0) had a significant effect on ENG (T1) (*β* = 0.11, *p* = 0.011), and TR (T1) had a significant effect on ENG (T2) (*β* = 0.10, *p* = 0.012). Contrary to our expectation, H3 was not supported by the data: ENG (T0) did not affect AUT (T1) (*β* = 0.04, *p* = 0.449), and ENG (T1) did not affect AUT (T2) (*β* = 0.04, *p* = 0.451). H4 was supported by the data, ENG (T0) had a significant effect on TR (T1), (*β* = 0.12, *p* = 0.007), and ENG (T1) had a significant effect on TR (T2), (*β* = 0.11, *p* = 0.009). Regarding the Improvement in private life, H5 seems to be confirmed: TR (T0) had a significant effect on IPL (T1) (*β* = 0.16, *p* = 0.003), and TR (T1) had a significant effect on IPL (T2) (*β* = 0.14, *p* = 0.005). On the contrary, H6 has not been confirmed from the data: AUT (T0) had a non-significant effect on IPL (T1) (*β* = 0.04, *p* = 0.456), and AUT (T1) had a non-significant effect on IPL (T2) (*β* = 0.04, *p* = 0.454). Covariates effects are reported in Table 4.

**Table 4:** Standardized effects of covariates on each variable of the cross-lagged panel model.

Effects	β	SE	p
Gender (T0) → TR (T0)	0.053	0.075	0.475
Children (T0) → TR (T0)	0.067	0.071	0.344
Gender (T0) → ENG (T0)	−0.034	0.070	0.627
Children (T0) → ENG (T0)	−0.052	0.076	0.497
Gender (T0) → AUT (T0)	−0.012	0.070	0.862
Children (T0) → AUT (T0)	−0.022	0.071	0.760
Gender (T0) → TR (T1)	−0.017	0.054	0.752
Children (T0) → TR (T1)	0.081	0.062	0.192
Gender (T0) → ENG (T1)	0.008	0.056	0.892
Children (T0) → ENG (T1)	−0.030	0.055	0.581
Gender (T0) → AUT (T1)	**−0.113**	**0.056**	**0.042**
Children (T0) → AUT (T1)	−0.028	0.065	0.668
Gender (T0) → IPL (T1)	0.073	0.081	0.365
Children (T0) → IPL (T1)	0.128	0.068	0.061
Gender (T0) → TR (T2)	−0.007	0.064	0.916
Children (T0) → TR (T2)	0.103	0.058	0.073
Gender (T0) → ENG (T2)	0.056	0.063	0.372
Children (T0) → ENG (T2)	0.077	0.054	0.155
Gender (T0) → AUT (T2)	−0.066	0.061	0.280
Children (T0) → AUT (T2)	−0.048	0.064	0.466
Gender (T0) → IPL (T2)	0.022	0.072	0.754
Children (T0) → IPL (T2)	0.095	0.075	0.202

TR, Technology as a resource; ENG, Work Engagement; AUT, Autonomy; IPL, Improvement in private life; Results are presented in a completely standardized metric. T0 = Time 1, T1 = Time 2, T2 = Time 3, B = Estimate; SE = Standard Error.

Significant coefficient are reported in bold.

Regarding the indirect effects, H7 was supported: AUT (T0) had a significant effect on TR (T2) through ENG (T1) (*β* = 0.01, *p* = 0.049; 95% CI = 0.002; 0.042). Finally, also H8 was confirmed: ENG (T0) showed a significant effect on IPL (T2) through TR (T1) (*β* = 0.02, *p* = 0.050; 95% CI = 0.002; 0.052). Regarding the role of covariates, the results showed that females scored lower in AUT at T1 (*β* = −0.113, *p* = 0.042). Overall, the model explained 36.9% of the variance of TR, 33.7% of AUT, 42.2% of ENG, and 5.3% of IPL measured at T1, and 45.8% of TR, 42.1% of AUT, 64% of ENG, and 33.5% of IPL measured at T3. Significant indirect effects are reported in Table 5.

**Table 5:** Standardized specific indirect effects and their associated bootstrapped confidence intervals of the cross-lagged model.

Specific indirect effects	Estimate	*p*	95% Bootstrapped CI
AUT (T0) → ENG (T1) → TR (T2)	0.015	0.049	0.002–0.042
ENG (T0) → TR (T1) → IPL (T2)	0.017	0.050	0.002–0.052

TR, Technology as a resource; ENG, Work Engagement; AUT, Autonomy; IPL, Improvement in private life.

## Discussion

Due to the pandemic, remote work has become increasingly prevalent, becoming a necessary modality of work (2), and organizations were forced to provide technological resources for remote work due to the emergency (3). This sudden change in work arrangements has led to workers having to adapt to a new way of working from home, often without or with low prior preparation (2). Implementing remote work significantly impacts workers’ well-being and motivation (61). This study offers a comprehensive exploration of how technology and job autonomy influence work engagement and private life improvements in the context of remote work. A key strength lies in its longitudinal design, capturing the evolution of these dynamics over three distinct phases of remote work implementation.

Invariance analyses confirmed the strict invariance, along with significant increases in TR’s latent means between T0 and T1, and a decrease in ENG latent means between T1 and T2. The perception of technology as a resource may have increased between T0 and T1 because the introduction of technological tools that enabled workers to perceive technology as positive and useful to the performance of their work. In addition, the spread of the COVID 19 pandemic may have caused a slight decrease in remote worker engagement between T1 and T2 due to stay at home laws.

The data support almost all of our hypotheses (Table 6). According to our first hypothesis, job autonomy increased work engagement over time. In line with previous studies, our result supported that autonomy positively impacts employee engagement (34). Workers’ ability to decide on the manner, place, and time of performing tasks increases engagement with their work. Moreover, autonomy encourages creativity and makes it easier to adapt to change (35) by providing employees with more responsibility and accountability. When people are free to make decisions and manage their tasks, they tend to feel more committed to the outcomes. This sense of ownership often leads to greater dedication to their work, resulting in higher levels of engagement.

**Table 6:** Resume of study hypotheses.

Hypothesis		Supported
H1	Job autonomy will positively sustain work engagement over time.	Yes
H2	The perception of technology as a resource will positively sustain work engagement over time.	Yes
H3	Work engagement will positively influence job autonomy over time.	No
H4	Work engagement will positively influence the perception of technology as a resource over time.	Yes
H5	The perception of technology as a resource would positively predict improvements in workers’ private lives.	Yes
H6	Job autonomy would positively predict improvements in workers’ private lives.	No
H7	Work engagement will mediate the relation between job autonomy and the perception of technology as a resource.	Yes
H8	The perception of technology as a resource will mediate the relation between work engagement and Improvement in private life.	Yes

Similarly, results confirmed our second hypothesis: technology as a resource positively influenced work engagement over time. When organizations implement effective practices and policies, such as providing adequate technological tools, remote workers can have a healthy and fulfilling work environment. For example, improving tools for sharing information and documents can help workers perform better and maintain contact with their colleagues (e.g., by using videoconference platforms). Nonetheless, when changes are introduced within the organization, clear communication and good sharing from management have been found to boost individual outcomes (17).

The results did not support our third hypothesis: work engagement was not significantly associated with job autonomy over time, possibly because the link between work engagement and job autonomy may depend on contextual or structural factors not captured in this study, such as team processes or managerial practices. Another explanation could reside in both the ways and the context in which workers have come to deal with remote work. Indeed, at T1, the workers were in remote work conditions 1 day per week. This innovative and different way of working may have prevented the workers from fully adapting to and experiencing the degree of autonomy provided by remote work. Thus, engagement may not have been able to influence autonomy in workers. On the other hand, at T2, workers were in full-time remote work (5 days per week) because of the lockdown due to the COVID-19 pandemic. Restrictions imposed by the government forced workers to stay at home, with very few opportunities to go out and perform activities outside their homes. This may have undermined workers’ sense of autonomy, so their work engagement may not have been able to stimulate feelings of autonomy fully.

As expected, work engagement positively influenced the perception of technology as a resource over time (H4). According to the JD-R model (10), more engaged workers may experience a positive cycle in which they become more engaged and motivated and perceive resources more positively. This can result in a gain spiral, in which the relationships between work engagement, proactive behavior and resources become more and more positive, leading to even higher levels of these variables [see also (37, 38)]. Thus, highly engaged workers had a more positive perception of the technology provided by their organization as a resource.

Our fifth hypothesis was supported by the data: the perception of technology as a resource significantly predicted the perception of Improvement of private life due to remote work overtime. Workers can use technology as a resource to improve their personal and work lives, including maintaining relationships with colleagues and loved ones (42). H6 was not supported by our results: job autonomy did not significantly predict an improvement in private life due to remote work over time, which may suggest that job autonomy alone is not sufficient to produce improvements in private life. Other elements such as individual coping strategies or external demands may play a more significant role. The challenges posed by the COVID-19 pandemic (T2) and adopting a new work model for just 1 day per week (T1) may have made it harder for participants to perceive any improvements in their lives resulting from working remotely. As a result, it is possible that autonomy had a smaller impact on their perception of remote work’s benefits.

The data supported our seventh hypothesis. Results showed that job autonomy prospectively influenced the perception of technology as a resource through the mediating effect of work engagement. Research shows that allowing workers to make decisions and control their work is vital for their engagement (34). Engaged workers tend to be more receptive to new experiences (43), which could make them more likely to view the technology provided by the organization as a valuable resource. Finally, H8 was supported by the data. Work engagement prospectively influenced the Improvement in private life due to remote work through the mediating effect of the perception of the technology provided by the organization as a resource. As per the JD-R (10) model, resources play a crucial role in promoting the well-being of employees. Therefore, when technology is framed as a resource, it can enhance the perception of well-being among workers. Likewise, more engaged employees are more open to novelty, perceiving the technologies provided by their organization as a resource. Hence, it is possible to assume that more engaged workers may experience a better quality of life due to technological resources.

### Limitations and future developments

The study conducted has yielded some significant results, but it also has limitations that need to be acknowledged. Firstly, all the participants were Italian and worked at INAIL, a very specific organization with its own culture, which may differ from that of other public or private sectors. This means that the results cannot be generalized to other countries or organizational contexts. Future studies should aim to replicate these findings with different samples and in diverse work contexts (e.g., private sector, multinational, younger workforce), to gain a more in-depth understanding of remote work and technology use. Second, the reliance on self-reported measures introduces potential bias, as the common method variance (CMV). Although procedural remedies were adopted to mitigate this risk, future research should consider triangulating self-reports with objective or behavioral data to enhance the robustness of findings. This would help address concerns related to CMV (62). Thirdly, the high average age of participants may have influenced their perspectives on technology and remote work, since older people tend to have more difficulty managing the use of new technologies, suggesting the need for research that includes a broader demographic range. Finally, we acknowledge the limitation of using a single-item measure for “improvements in private life,” which was due to survey length constraints. It will be helpful to consider validated multi-item scales to enhance measurement reliability. Future studies should also investigate long-term effects of remote work on engagement, well-being, and private life, considering evolving technological advancements and organizational policies. By addressing these limitations, future studies can provide deeper insights and practical guidelines for optimizing remote work practices.

## Conclusion and practical implications

Remote work has become increasingly popular due to the benefits of saving on physical workspace, access to a wider pool of global talent, and less time spent commuting (7). However, it must be properly introduced in order to prevent negative outcomes. Introducing technological tools in public administration for remote work can help overcome remote workers’ unique challenges. These tools can enhance communication and collaboration among remote workers, providing efficient channels for them to connect with colleagues, share information, and collaborate on projects (63). By offering real-time messaging platforms, video conferencing capabilities, and project management tools, new technological tools can improve communication and coordination between team members, even when they are physically distant. Implementing new technological tools in public administration for remote work has the potential to positively transform the way remote workers collaborate and execute tasks, leading to an enhancement in their private lives. It is essential for organizations to tailor their remote work programs to the specific needs of their workforce, taking into account factors such as job roles, communication needs, and team dynamics. Providing adequate support for remote workers, including flexible work practices and addressing potential challenges such as social isolation, is crucial for the success of remote work arrangements. Overall, by understanding and addressing these practical implications, organizations can create successful and sustainable remote work and job design strategies.

## Data Availability

The data analyzed in this study is subject to the following licenses/restrictions: the dataset generated and analyzed in this study is not publicly available due to institutional restrictions and confidentiality agreements with the participating organization (INAIL). Access to the data may be granted upon reasonable request and with permission from INAIL and the research team. Requests to access these datasets should be directed to Ilaria Olivo, ilaria.olivo@uniroma1.it.

## Footnotes

^1^The short Italian version of the Management Standards Indicator tool was developed by INAIL in collaboration with the Department of Psychology of Sapienza University of Rome. The “optimal shortening” procedure (64), which allows to maximize the internal coherence of the tools without sacrificing the representativeness of the content of the items with respect to the factor they measure, was used to obtain a short version of this tool. A validation study on this short version is underway.
